# Does tumor rupture during robot-assisted partial nephrectomy have an impact on mid-term tumor recurrences?

**DOI:** 10.2478/raon-2023-0031

**Published:** 2023-07-20

**Authors:** Simon Hawlina, Kosta Cerovic, Andraz Kondza, Peter Popovic, Jure Bizjak, Tomaz Smrkolj

**Affiliations:** Clinical Department of Urology, University Medical Centre Ljubljana, Ljubljana, Slovenia; Department of Surgery, Faculty of Medicine, University of Ljubljana, Ljubljana, Slovenia; Clinical Institute of Radiology, University Medical Centre Ljubljana, Ljubljana, Slovenia; Faculty of Medicine, University of Ljubljana, Ljubljana, Slovenia

**Keywords:** enucleation, tumor recurrence, renal cell carcinoma, robot-assisted partial nephrectomy, tumor rupture, warm ischemia time

## Abstract

**Background:**

Intraoperative kidney tumor rupture (TR) can occur during robot-assisted partial nephrectomy (RAPN) in daily clinical practice, but there are no solid guidelines on the management and implications of it. The purpose of the study was to investigate the impact of TR on tumor recurrences, what a surgeon should do if this adverse event occurs, and how to avoid it.

**Patients and methods:**

We retrospectively analyzed the first 100 patients who underwent RAPN at University Medical Centre Ljubljana, between 2018 and 2021. Patients were stratified into 2 groups (TR and no-TR) and were compared according to patient, tumor, pathologic, perioperative and postoperative characteristics and tumor recurrences, using the Mann-Whitney U test and chi-squared test.

**Results:**

Of the 100 patients, 14 had TR (14%); this occurred in tumors with higher RENAL nephrometry scores (P = 0.028) and mostly with papillary renal cell carcinomas (P = 0.043). Median warm ischemia time was longer for the TR group (22 *vs.* 15 min, *P* = 0.026). In terms of studied outcomes, there were no cases of local or distant recurrence after a median observation time of 39 months (interquartile range, 31–47 months) in both groups. We observed positive surgical margins on the final oncologic report in one case in the no-TR group.

**Conclusions:**

Tumor rupture during RAPN seems to be of no mid-term oncologic importance. According to presented results, we would recommend surgeons to proceed with tumor resection if this event occurs and abstain from conversion to radical nephrectomy or open partial nephrectomy. However, more similar cases should be studied to make more solid conclusions.

## Introduction

Partial nephrectomy (PN) is the treatment of choice for T1 renal cell carcinoma (RCC) because it provides comparable oncological safety while better preserving renal function, thus leading to a lower incidence of cardiovascular diseases.^1^ Tumor enucleation is a safe procedure oncologically (perioperative, short-term, and long-term) when negative surgical margins are achieved by providing a microscopic layer of healthy kidney tissue on the surface of the tumor.^2,3,4^

However, decreased distance between healthy parenchyma and the tumor pseudocapsule increases the risk of slitting into the tumor (positive surgical margin) or even rupturing the tumor during excision and tumor manipulation (tumor cell spillage). There is no clear definition of TR or so-called accidental slit into the tumor with consequent spillage of tumor cells into the operative field and abdominal cavity, the frequency of which has been underestimated and the clinical impact insufficiently investigated in the literature.^5,6,7^ One simple inattentive move with sharp instrument by surgeon or assistant could disrupt already thin layer left on the surface of the tumor. Obviously, this would happen less frequently if more of the healthy tissue is left over the tumor capsule.

It has been known that a positive surgical margin in a low malignant tumor does not necessarily lead to recurrence of the disease but there is a higher chance of recurrence in tumors with higher malignant potential.^8^ On the other hand, a little is known if macroscopic spillage of the tumor cells occurs.^5,6,7^ The purpose of this study was to investigate the rate of tumor recurrences and clinical impact of tumor rupture (TR) during robot-assisted partial nephrectomy (RAPN), what a surgeon should do in the case of this undesired event and how to avoid it. The rate of tumor recurrences was measured with radiological evidence of tumor in the locoregional region and abdominal cavity.

## Patients and methods

### Study design and surgical technique

We conducted a retrospective study of the first 100 patients who underwent RAPN at University Medical Center (UMC) Ljubljana between June 2018 and April 2021. RAPN was performed by 2 senior surgeons, who had a previous experience in both open and laparoscopic partial nephrectomies. Our detailed technique of transabdominal RAPN has been described previously.^9^ A transperitoneal approach was used in 90 procedures (90%) and a retroperitoneal approach was used in 10 procedures (10%). In 8% of cases, we removed two tumors during the same procedure. In these cases, a comprehensive standardized system for quantitating renal tumor size, location and depth (RENAL) score^10^ and final histology were determined only for the larger tumor. We always try to perform enucleation of the tumor, aiming for maximal preservation of healthy renal parenchyma and renal function. No frozen sections were performed during RAPN.

Medical Ethics Committee of the Republic of Slovenia approved this study (registration number 0120-68/2023/3) and it was conducted in full compliance with the principles of the Declaration of Helsinki.

**FIGURE 1.**
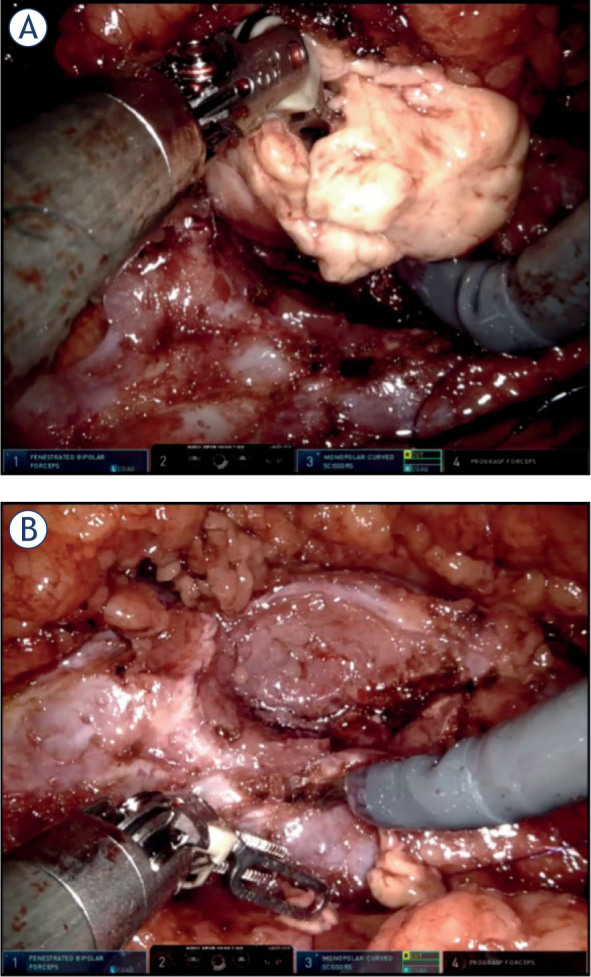
**(A)** Example of tumor rupture during enucleation of a renal tumor. **(B)** Tumor bed after the tumor was completely removed from healthy kidney parenchyma (intraoperative snapshots).

TR was defined as an intraoperative (macroscopic) slit into a tumor during tumor resection and/or tumor manipulation, which could lead to spillage of the tumor cells into the operative field and the abdominal cavity (Figure 1). Our definition is based on the definition by Khene *et al.* who defined accidental surgical incision into the tumor (ASIT) as “any accidental incision in the tumour or any accidental rupture of tumour surface during handling of the kidney and/or tumor”.^5^ We want to emphasize a clear distinction between TR (an intraoperative, macroscopic event) and positive surgical margins (a histologic, postoperative, microscopic event).

Recurrence was defined as local recurrence at the enucleation site or atypical intraabdominal locations^2^, observed on follow-up contrast-enhanced computed tomography (CT).

Patients were divided into 2 groups: tumor rupture (TR) and no tumor rupture (no-TR). Our null hypothesis was that the TR can occur independently of the radiologic, pathologic, or intraoperative variables, so all 100 patients were included in the study.

### Postoperative follow-up regimen

The follow-up was performed by the urologists; the scheme depends on the tumor characteristics (size, histology, grade, resection margin, TNM classification, etc.) and the patient's life expectancy. All patients underwent regular cross sectional imaging – we followed recommendations for surveillance proposed by EAU guidelines.^1^ For the purpose of the study, an additional contrast-enhanced CT was performed in all 14 cases of TR in May 2022. All CT reviews were performed by 2 abdominal radiologists (with more than 10 years of experience in kidney imaging), blinded to all clinical, biological and follow-up data.

### Statistical analysis

The Mann-Whitney U test was used for analysis of continuous variables, presented as medians and interquartile ranges (IQRs). The chi-squared test was used to determine the relationship between categorical variables, presented as proportions. Both tests were two-sided and the significance level was set at *P* < 0.05.

## Results

### Patient and tumor characteristics

The characteristics of the patients who underwent RAPN at UMC Ljubljana between June 2018 and April 2021 are shown in Table 1. The median duration of follow-up was 39 months (IQR, 31–47 months). TR occurred in 14 cases. In the TR group, tumors tended to be larger (37 mm *vs.* 30 mm) and had a higher RENAL score (8 *vs.* 7); only the latter reached statistical significance (*P* = 0.028). Both groups were comparable in terms of sex (*P* = 0.8), median age at surgery (*P* = 0.9), tumor laterality (*P* = 0.8), and localization (*P* = 0.8).

**TABLE 1.** Patient and tumor characteristics in the no tumor rupture group and the tumor rupture group

	**No tumor rupture (*N* = 86)**	**Tumor rupture (*N* = 14)**	***P* value**
Patients, *n* (%)			
Male	59 (69)	9 (64)	0.8
Female	27 (31)	5 (36)	
Age (years), median (IQR)	60 (52–67)	60 (49–68)	0.9
Tumor size (mm), median (IQR)	30 (23–40)	37 (30–48)	0.2
RENAL nephrometry score, median (IQR)^*^	7 (5–8)	8 (6.25–9.75)	**0.028**
Laterality, *n* (%)			
Right kidney	40 (47)	6 (43)	0.8
Left kidney	46 (53)	8 (57)	
Tumor localization, *n* (%)			
Upper third	24 (28)	5(36)	0.8
Middle third	34 (39)	5 (36)	
Lower third	28 (33)	4 (28)	
Preoperative CT/MRI, *n* (%)			
Tumor	72 (84)	13 (93)	0.4
Cystic	14 (16)	1 (7)	

Bold indicates a significant value (*P* < 0.05).

* RENAL score was determined for 82 of 86 tumors, because 4 CT scans were not available for interpretation.

CT = computed tomography; IQR = interquartile range; MRI = magnetic resonance imaging

### Pathological characteristics and oncologic outcomes

Pathologic characteristics and oncologic outcomes are summarized in Table 2. RCC was identified in 83 patients, oncocytoma in 8, and benign tumors in the remaining 9 cases. The most frequent histologic type was clear cell RCC (ccRCC) (46%), followed by papillary RCC (pRCC) (23%). ccRCC was significantly more frequent in the no-TR group (51% *vs.* 14%), whereas pRCC was the most common type in the TR group (57% *vs.* 17%, *P* = 0.043). Type I pRCC was more frequent than type II pRCC in both groups (7 of 8 [88%] in the TR group *vs*. 13 of 15 [87%] in the no-TR group). There was no statistically significant difference between the groups regarding tumor grade (*P* = 0.6), pathologic stage (*P* = 0.4), and positive surgical margins (*P* = 0.7). Most of the tumors were pT1a (82% in the no-TR group *vs.* 75% in the TR group). No cases of tumor recurrences were observed.

**TABLE 2.** Pathologic characteristics and oncologic outcome in the no tumor rupture group and the tumor rupture group

	**No tumor rupture (*N* = 86), *n* (%)**	**Tumor rupture (*N* = 14), *n* (%)**	***P* value**
Histology			**0.043**
Benign	8 (9)	1 (7)	
Oncocytoma	7 (8)	1 (7)	
Clear cell RCC	**44 (51)**	2 (14)	
Papillary RCC	15 (17)	**8 (57)**	
Chromophobe RCC	5 (6)	1 (7)	
Clear cell papillary RCC	4 (5)	0 (0)	
Other types of RCC	3 (3)	1 (7)	
WHO/ISUP grade (RCC)			0.6
1	21 (35)	2 (25)	
2	35 (58)	6 (75)	
3	4 (7)	0 (0)	
Pathologic stage			0.4
1a	58 (82)	9 (75)	
1b	9 (13)	2 (17)	
2a	1 (1)	1 (8)	
3	3 (4)	0 (0)	
Positive surgical margins	1 (1)	0 (0)	0.7
Local or distant recurrence	0 (0)	0 (0)	

Bold indicates a significant value (*P* < 0.05).

ISUP = International Society of Urologic Pathologists; RCC = renal cell carcinoma; WHO = World Health Organization

### Perioperative and postoperative outcomes

Perioperative and postoperative outcomes are summarized in Table 3. The median duration of the surgical procedure (147 min *vs.* 140 min, *P* = 0.4) and the median hospital stay after the operation (3 days *vs.* 3 days, *P* = 0.8) were not significantly different in the TR and no-TR groups. Median WIT was significantly longer in the TR group (22 *vs.* 15 min, *P* = 0.026). Median estimated blood loss was higher in the TR group (50 *vs.* 20 mL), but the result did not reach statistical significance (*P* = 0.13).

**TABLE 3.** Perioperative and postoperative outcomes in the no tumor rupture group and the tumor rupture group

	**No tumor rupture (*N* = 86)**	**Tumor rupture (*N* = 14)**	***P* value**
Operative time (min), median (IQR)	140 (115–171)	147 (135–168)	0.4
WIT (min), median (IQR)	15 (12–19)	22 (15–25)	**0.026**
No clamping, *n* (%)	8 (9)	0 (0)	
Length of stay after surgery (days), median (IQR)	3 (2–3)	3 (2–3)	0.8
Creatinine (μmol/L), median (IQR)			
Preoperative	80 (73–94)	80 (77–87)	0.9
2 days after RAPN	80 (70–98)	80 (75–89)	0.6
Variation	1 (−7 to 7)	1 (−7 to 8)	0.7
Intraoperative EBL (mL), median (IQR)	20 (0–50)	50 (20–100)	0.13
Hemoglobin (g/L), median (IQR)			
Preoperative	148 (140–155)	148 (144–152)	0.7
2 days after RAPN	125 (119–133)	125 (122–129)	0.8
Variation	22 (14–27)	25 (20–27)	0.6
Transfusions, *n* (%)	3 (3)	0	
Major complications (Clavien-Dindo ≥ 3), *n* (%)	2 (2)	0	
Conversions to radical nephrectomy, *n* (%)	2 (2)	0 (0)	

Bold indicates a significant value (P < 0.05).

EBL = estimated blood loss; IQR = interquartile range; RAPN = robot-assisted partial nephrectomy; WIT = warm ischemia time

Nine percent of procedures in the no-TR group and none in TR group were performed with the no clamping method. We performed two conversions to radical nephrectomy; once due to an ipsilateral incidentaloma not seen on preoperative CT imaging and once due to the size of the tumor, which had increased significantly since the preoperative CT. There were no conversions to open surgery.

The median creatinine level preoperatively and postoperatively and the change in creatinine were comparable between the 2 groups; it stayed near the preoperative level. Similarly, the median hemoglobin level preoperatively, postoperatively, and the median decrease in hemoglobin did not significantly differ between the groups; the median decrease was 22 g/L in the no-TR group and 25 g/L in the TR group (*P* = 0.6).

Three patients in the no-TR group needed blood transfusions after the procedure. We observed 2 major complications (defined as Clavien-Dindo classification score 3 or more^11^); one required exploration due to bleeding from the vessel at the umbilical port position and the other required superselective embolization due to active bleeding from a small renal artery branch in the tumor bed.

## Discussion

To the best of our knowledge, there have been only a few papers investigating the effect of tumor rupture or cyst rupture during robotic PN.^5,6,7^ On the other hand, a positive surgical margin is much more widely researched and discussed. It seems that a positive surgical margin in cases of RCC (especially of low grade and size) is not associated with an increased risk of recurrence or decreased survival rates as opposed to transitional cell carcinomas or adrenocortical carcinomas.^12,13,14,15^ In the context of surgical margin assessment, it is debatable if only TR of the bottom border of the tumor is relevant as rupture can occur far from healthy parenchyma interface. In that case, a surgeon could make a complimentary resection of the tumor bed, so minority of TRs result in a positive surgical margin. In addition, TR can occur when a surgeon or an assistant makes a macroscopic slit into the tumor or a tumor breaks because of manipulation during excision.

In our study, we observed 14 cases of intraoperative TR (14%), which is a high number, especially for something not usually reported in the literature. After a median of 39 months (IQR, 31–47 months), we recorded no cases of tumor recurrence. Interestingly, Khene *et al.* showed the same percentage of accidental surgical incision into the tumor (ASIT) as we did and concluded it as “common event that did not appear to compromise oncological outcome”.^5^ They observed 9% of recurrences in the ASIT group and 6% in the control group after median follow-up 36 months, while nearly 43% of their cases were high risk tumors (pT2–3A and/or Fuhrman Grade III–IV)^5^ as opposite to ours. Takagi *et al.* also showed high tumor grade along with pathological tumor upstaging from cT1 to pT3 to be risk factors for worse recurrence-free survival.^16^ In addition, Grossmann *et al.* presented a case report of peritoneal carcinomatosis of the cystic papillary renal cell carcinoma following intraoperative cyst rupture during partial nephrectomy.^17^ Apart from early recurrence, there is also a possibility of late recurrence (recurrence after 5 years) which occurs in around 3.5%; main predictive factors for it are higher pathological stage (≥ pT2) and age at surgery.^18,19^

Among 14 cases of TR in our study, 86% were carcinomas, 7% were oncocytomas, and 7% were benign tumors, all of them were included because we did not want to solely investigate recurrences. One multicenter cohort study reported an 18.7% rate of intraoperative cystic renal masses rupture *via* an open or robot-assisted approach, which had no influence on tumor recurrences, including no cases of local or distal recurrences.^6^ Another group identified risk factors for cystic RCC rupture to be higher E (exophytic/endophytic) and N (nearness to collecting system or sinus) RENAL nephrometry scores, higher Bosniak category (specifically III), and surgeon's experience.^7^ Even though recurrence-free survival and cancer-free survival were worse if cystic RCC rupture occurred, it did not seem to influence overall survival.^7^

The only study that indeed investigated the impact of tumor rupture (in their paper called “effraction”) during RAPN showed the main determinants of accidental slit into the tumor to be size of the tumor and experience of the surgeon.^5^ According to our results, a high RENAL nephrometry score seems to be related to TR (*P* = 0.028). In a TR group, tumors tended to be larger (37 mm *vs.* 30 mm), but the result did not reach statistical significance (*P* = 0.2). With regard to surgeon experience, we observed a decrease in the number of TRs over time. In the first 20 cases, there were 5 (25%) TRs, but the percentage decreased to 11% in the following 80 procedures. However, this result did not reach statistical significance (*P* = 0.11). We suggest 3 reasons that could explain this: (1) with more experience, we started operating more difficult cases; (2) TRs also occur as a consequence of tumor manipulation by an assistant and are not solely dependent on the mistakes/experience of a surgeon; (3) due to a low number of cases, the results did not show the statistical significance. Tumor enucleation is more technically demanding, therefore it could be a risk factor for TR. It is an oncologically safe surgical technique whereby the surgeon leaves a microscopic layer of healthy kidney tissue on the surface of the tumor.^1,2,4^ Generally, results regarding recurrences at the enucleation site differ in the literature (ranging from 0% to 8%, depending on the size of the tumor, pT stage, RENAL nephrometry score, follow-up duration). Benign tumors and lower pT stage RCCs did not recur after the follow-ups, whereas RCCs with higher pT stage did.^20,21,22^ For example, in sporadic follow-up of RCCs of at least 4 years, there were no recurrences at the enucleation site.^2^ According to Minervini *et al.* positive surgical margins, recurrence in the ipsilateral kidney (either at the enucleation site or elsewhere), and systemic recurrence were all found in 2.4% of cases, and < 1% of patients died due to metastatic RCC after the median follow-up of 61 months.^2^ Similarly, Hu *et al.* observed positive surgical margins in 3.5% of cases and less than 1% of recurrences after a median follow-up of 2.7 years.^23^ We performed tumor enucleation in most cases and observed a positive surgical margin in 1 case, which is comparable with the results in the literature.^2,23^

We wanted to determine the influence of tumor type on the occurrence of tumor rupture. In our series, final pathology reports showed that most ruptured tumors were papillary RCCs, which is not surprising. Fragility is a typical feature of pRCC type I; this can be explained by its histology because its narrow papillae contain only microcapillaries without any binding and a tough pseudocapsule (specimens are described as a “minced meat” structure).^1^ Some studies show the peritumoral pseudocapsule to be less developed (thinner, incomplete, or absent) in pRCCs compared with ccRCCs.^24,25^ In addition, Hora *et al.* described 3 cases of spontaneous rupture of pRCCs or after minimal trauma due to extensive necrosis.^26^ Moreover, pRCCs have been shown to have a substantial risk of renal tumor biopsy tract seeding (12.5%), indicating its malignant potential.^27^ However, we did not observe tumor recurrence in any of the cases in the TR group.

We also wanted to determine the impact of tumor rupture on the possibility of complications during and after surgery. Pradere *et al.* showed that intraoperative cyst rupture during PN led to more postoperative complications^6^,which were not observed in our study. Our results showed that duration of the surgical procedure, duration of hospital stay, creatinine and hemoglobin levels (preoperatively and postoperatively) did not significantly differ between the TR and no-TR groups. Even though the estimated blood loss was higher in the TR group (50 *vs.* 20 mL, *P* = 0.13), the decrease in hemoglobin was not significantly different between the groups (25 *vs.* 22 g/L, *P* = 0.6). We observed 2 major complications (defined as Clavien-Dindo classification score 3 or more), but only in the no-TR group. On the other hand, WIT was significantly longer in the TR group, which could be explained in 3 ways: (1) tumor rupture with spillage of tumor tissue impairs visibility, resulting in more difficult tumor manipulation and further resection; (2) the surgeon decides to perform complementary resection of the tumor bed; (3) psychological stress experienced by the surgeon and decision making on how to proceed with the surgery. Interestingly, there was no case of tumor rupture within the no clamping group, which shows that bleeding during tumor resection alone with impaired visibility is not a sufficient reason for TR.

According to all these findings, we suggest that the surgeon should be careful to avoid TR when performing enucleation of kidney tumors. If pRCC is expected, we suggest enucleoresection instead of enucleation. The surgeon should always warn the assistant to be equally careful with any tumor manipulation (e.g., suction), especially if the tumor seems fragile. It is important that the surgeon stays focused and calm if TR occurs. Clear communication in the team is essential. The surgeon should assess the ability to control bleeding and extent of the spillage of the tumor cells, followed by the decision whether to convert to radical nephrectomy or even to open procedure for better visualization and control. If the surgeon decides to continue robot-assisted approach, sufficient irrigation of the surgical field and consequent suction are needed in order to remove spilled tumor cells. Moreover, a change in strategy (reduction of pneumoperitoneum pressure or switching to global ischemia) should also be considered. It is advisable to require the assistance of more experienced colleagues. After the procedure, patient documentation should be presented at the multidisciplinary team meetings in order to discuss potential adjuvant therapy or follow-up procedures and imaging. We believe that usage of three-dimensional models could make enucleations easier and decrease rates of surgical injury to the tumor.^28^

There are a few limitations of our study. First, the median follow-up of 39 months is relatively short to observe local recurrences, even though in the study by Khene *et al.* they observed recurrences after nearly equal follow-up.^5^ Moreover, in the study by Takagi *et al.* median time from PN to recurrence was 19 months.^16^ Second, the definition of TR is questionable because there is no clear pathologic-surgical agreement on what TR is, therefore we used the one available in the literature.^5^ Third, due to the retrospective single-center design of the study, there is a possibility of biased interpretation of the results.

## Conclusions

TR is a possible complication during RAPN, especially if tumor enucleation is performed on pRCCs with a higher RENAL nephrometry score, leading to prolonged WIT. We suggest proceeding with the resection of the tumor with a deeper resection plane and only eventually converting to radical nephrectomy or open PN, because it seems that TR has no mid-term risk of tumor recurrence or higher complication rate. The rate of long term effects of TR on tumor recurrences are still unknown.
